# CLN3 clinches lysosomes in clearance of glycerophospholipids

**DOI:** 10.1093/lifemeta/loac029

**Published:** 2022-10-19

**Authors:** Guang Lu, Han-Ming Shen

**Affiliations:** Department of Physiology, Zhongshan School of Medicine, Sun Yat-sen University, Guangzhou, Guangdong 510080, China; Department of Biomedical Sciences, Faculty of Health Sciences, Ministry of Education Frontiers Science Center for Precision Oncology, University of Macau, Macao 999078, China

CLN3 is a lysosomal transmembrane protein and loss of *CLN3* is known to cause a juvenile lethal neurodegenerative lysosomal storage disorder (LSD), called Batten disease. In a recent study published in *Nature*, Laqtom *et al.* reported a novel function of CLN3 in the clearance of glycerophospholipid from lysosomes via lysosomal efflux of glycerophosphodiesters (GPDs), not only establishing a deeper mechanistic understanding of Batten disease, but also suggesting both the diagnostic and therapeutic potential of CLN3-GPDs in this type of neurodegenerative LSD.

Lysosomes are the digestive organelles featured with an acidic lumen that facilitates the functions of digestive enzymes (hydrolases), and are closely implicated in various cellular processes, including autophagy, endocytosis, phagocytosis, etc [1, 2]. In addition, lysosomes play critical roles in metabolism (as the main organelle for catabolism) and serve as a signaling node linking nutrients and the mechanistic target of rapamycin complex 1 (mTORC1) pathway [3]. Thus, lysosomal dysfunction has been closely implicated in human diseases including neurodegenerative diseases such as Alzheimer disease, Parkinson disease, and cancers, just to name a few. In particular, loss of function mutations in genes encoding lysosomal proteins cause severe disorders that are collectively known as lysosomal storage disorders (LSDs), a group of inherited metabolic disorders [4]. Therefore, understanding the molecular mechanisms controlling the lysosome function is critically important for development of novel therapeutic approaches for the above-mentioned diseases.

Up to date, there are >70 different types of LSDs reported, all caused by deficiencies of lysosomal function due to mutation of key lysosomal genes, and are featured by accumulation of specific contents inside the lysosomes, mainly lipids, glycoproteins, and mucopolysaccharides [4, 5]. The clinical symptoms of LSDs vary depending on the type of LSDs and the stage. Owning to the great efforts in mechanistic studies and development of therapeutic approaches, there are some therapies for certain specific types of LSDs, such as enzyme-replacement therapy, substrate reduction therapy, or chaperone therapy [6, 7]. Unfortunately, there is no effect cure for many types of LSDs, including Batten disease.

**Figure 1:**
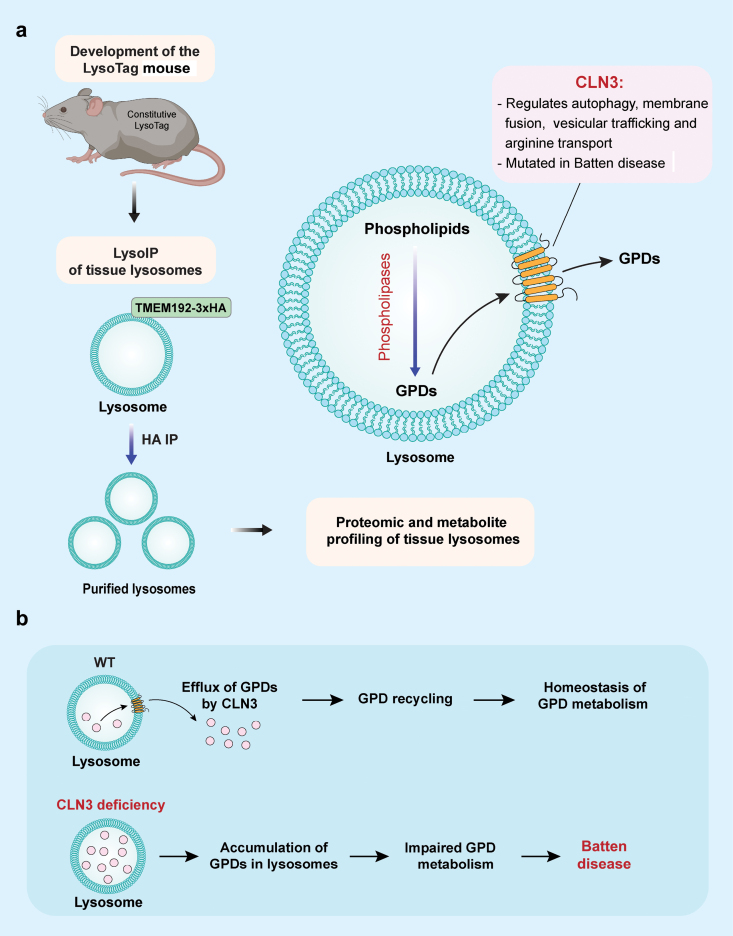
CLN3 links lysosomes to neurodegeneration via clearance of glycerophospholipids. (a) Schematic illustration of the steps for lysosome proteomic and metabolite analysis using LysoTag mouse. Briefly, mouse constitutively expressing the TMEM192-3xHA across tissues to mark lysosomes with a triple HA epitope and hence these lysosomes could be purified by immunoprecipitation with anti-HA antibodies. This process is termed LysoIP. Subsequently, these purified lysosomes could be subjected for proteomic and metabolite analysis. (b) Deficiency of CLN3 alters lysosomal metabolome. Under normal conditions, CLN3 promotes the efflux and recycling of GPDs to maintain the homeostasis of GPD metabolism. Upon CLN3 deficiency, GPD efflux from lysosome is blocked and hence GPDs accumulate in the lysosome, which impairs GPD recycling and lysosomal functions, and ultimately leads to Batten disease.

CLN3 is a multi-transmembrane protein that mainly localizes to the lysosome and is known to have multiple cellular functions, including autophagy, membrane fusion, vesicular trafficking, and transport [8]. Although it is well known that loss of function mutation of *CLN3* gene causes Batten disease, a type of neurodegenerative LSDs featured with vision loss, seizures, progressive dementia, motor function loss, and premature death, the exact role of CLN3 in regulating lysosomal functions remains to be fully addressed. In this study [9], the authors first established a transgenic mouse line, and this LysoTag mice constitutively express TMEM192–3×HA, a lysosomally localized fusion protein, across all tissues. The availability of this transgenic mouse line enables the authors to perform efficient immunoisolation to obtain pure and intact lysosomes from various tissues and cultured cells (Fig. 1a). Next, they focused on *CLN3*, a gene that is known to be mutated in Batten disease. To do this, they established a double transgenic line by crossing the LysoTag mice with *Cln3*^*−/−*^ mice and performed untargeted lipidomics analysis in brain lysosomes. They found that loss of *CLN3* led to abnormal lysosomal metabolome in brain, featured by significant increase of lysosomal lipids, inclusive of various glycerophospholipids. Principal component analyses together with mass spectrometry imaging (MSI) and MS/MS indicates that CLN3 mainly affects a set of glycerophosphodiesters (GPDs) in lysosomes: glycerophosphoglycerol, glycerophosphocholine, glycerophosphoinositol (GPI), and glycerophosphoethanolamine, which represent four of the five major GPDs. Similar results were also found in *CLN3*-KO HEK293T cells. Importantly, there was marked increase of GPI levels in the cerebrospinal fluid from patients with Batten disease, a phenomenon only found in Batten disease, but not in two disease models, thus suggesting the diagnostic value of this index. Moreover, the authors also recaptured the similar phenotypes in yeast with deletion of its yeast homologue of CLN3, suggesting that the function of CLN3 in GPD metabolism is evolutionarily conserved. In the final part of the study, the authors examined the molecular mechanisms for the increased GPDs in CLN3-null lysosomes. Theoretically, there are two possibilities: due to reduced hydrolysis of GPDs into glycerol 3-phosphate and their corresponding headgroups or due to impaired lysosomal efflux of GPDs. Via an array of elegant assays, the authors found that CLN3 specifically mediates efflux of GPDs from lysosome without altering the lysosomal pH, nor the efflux of amino acids (Fig. 1b).

Although this novel discovery correlates CLN3 to GPD metabolism, there are several important questions remaining to be further addressed. First, it is not known how the accumulating GPDs in lysosome would impair the lysosomal functions and eventually cause the phenotypes of Batten disease. Second, there are no data showing the link between CLN3-mediated-GPD metabolism and the neurodegenerative phenotypes of the CLN3-null mice. Lastly, for Batten disease patients, the authors found increased level of GPI in their cerebrospinal fluid, then it would be of interest to understand how those GPDs are released from lysosomes into cerebrospinal fluid in the absence of a functional CLN3.

In summary, in this study, by using the *in vivo* LysoIP method in combination with untargeted metabolite profiling, the authors presented convincing evidence that CLN3 is a key regulator in control of GPD efflux from lysosome. Thus, CLN3-mediated-GPD metabolism could be the legitimate targets for development of potential therapeutic approaches for this type of neurodegenerative LSDs.
